# The Function of Fucosylation in Progression of Lung Cancer

**DOI:** 10.3389/fonc.2018.00565

**Published:** 2018-12-07

**Authors:** Liyuan Jia, Jing Zhang, Tianran Ma, Yayuan Guo, Yuan Yu, Jihong Cui

**Affiliations:** ^1^Laboratory of Tissue Engineering, College of Life Sciences, Northwest University, Xi'an, China; ^2^Laboratory for Functional Glycomics, College of Life Sciences, Northwest University, Xi'an, China; ^3^Key Laboratory of Resource Biology and Biotechnology in Western China Ministry of Education, Xi'an, China; ^4^Provincial Key Laboratory of Biotechnology of Shaanxi, Xi'an, China

**Keywords:** lung cancer, fucosylation, fucosyltransferase, biomarker, epithelial–mesenchymal transition

## Abstract

Lung cancer is a disease that influences human health and has become a leading cause of cancer mortality worldwide. However, it is frequently diagnosed at the advanced stage. It is necessary by means of biology to identify specific lung tumor biomarkers with high sensitivity. Glycosylation is one of the most important post-translational modifications and is related to many different diseases. It is involved in numerous essential biological processes, such as cell proliferation, differentiation, migration, cell-cell integrity and recognition, and immune modulation. However, little was known about deregulation of glycosylation in lung cancer and contribution to tumor–microenvironment interactions. Among the numerous glycosylations, fucosylation is the most common modification of glycoproteins and glycosylated oligosaccharides. Increased levels of fucosylation have been detected in various pathological conditions, as well as in lung cancer. In this article, we reviewed the role of fucosylation in lung cancer. We highlighted some of the fucosylation alterations currently being pursued in sera or tissues of lung cancer patients. Moreover, we elaborated on the regulation mechanism of fucosylation in proliferative invasion and metastasis of lung tumor cells. In summary, alterations in fucosylation provide potential biomarkers and therapeutic targets in lung cancer.

## Introduction

Lung cancer, a primary cause of death, has increasing incidence and mortality rates worldwide (1–3). According to the World Health Organization (WHO) classification (4–6), lung cancer is categorized into small cell lung cancer (SCLC) and non-SCLC (NSCLC), which can be further subdivided into three major categories: adenocarcinomas (ADCs), squamous cell carcinomas (SQCCs), and large cell carcinomas (LCs). Because of the early stage patients not obvious symptom, most patients with lung cancer are diagnosed in the terminal stage and are estimated to have a survival time lower than 5 years. Now commonly used the methods for early diagnosis of lung cancer are Magnetic Resonance Imaging (MRI), Positron Emission Tomography-Computed Tomography (PET-CT), Endo-Bronchial Ultra-Sound (EBUS), Endocytoscopy (EC), Transbronchial Needle Aspiration (TBNA), and combined Testing of Serum Tumor Markers et al. However, there is a favorable survival rate in patients with earl stage lung cancer. As a result, searching for new biomarkers with high sensitivity and specificity to lung cancer will provide us with critical evidence in early diagnosis, therapy guidance, and prognosis monitoring. It has significance in increasing lung cancer survivability.

Aberrant glycosylation occurs frequently during carcinogenesis. The occurrence and development of malignant disease is accompanied by glycosylation (7). Most of the secreted and cell-surface proteins are glycoproteins which contain different types of oligosaccharide glycans covalently attached to amino acid side-chains are modified by different types of glycan structures. Glycosylation is orchestrated by numerous glycosyltransferases and glycosidases. The *N*- and *O*-linked glycan structures of glycoproteins serve different functions related to cell-cell communication. By using systemic glycomics approaches, we can further understand the functional roles of glycansin under physiological and pathological conditions (8). The alteration of the glycan number and structure are tightly related to cancer progression, invasion, metastasis, and epithelial–mesenchymal transition (EMT) in cancer cells (9–11). Studies suggested that factors like sex, age, and blood group are related to glycosylation (12–15). The age- and sex-associated differences in the glycopatterns of human glycoproteins may help us understand some age related to diseases and physiological phenomena specific to women or men. But researches about the glycosylation in different cancer patients with different sex, age, and blood group are seldom. More attention should be paid to this filed. Some glycoproteins have been used as diagnostic indexes of cancer, such as alpha feto-protein (AFP) and carcinoembryonic antigen (16, 17). Extensive progress has been achieved in the research of the glycosylation changes in lung cancer in recent years, which provide references to the diagnosis and treatment of lung cancer. However, due to the pathogenesis underlying lung cancer is complicated, there are almost no corresponding application in clinical diagnosis and treatment. Identifying and characterizing distinctive glycosylation changes in lung cancer by means of proteomic methods is important for its early detection and the target therapy.

Fucosylation, a type of glycosylation, has been studied frequently. It participates in the biosynthesis of blood H antigen and Lewis antigen, leukocyte extravasation mediated by selectin, host-microbe interactions, and modification of signaling (3, 18). Fucosyltransferase mediates the transfer of fucose residues to oligosaccharides and/or proteins. In lung, breast, brain, liver, and thyroid cancer, differential expression of the fucosyltransferase enzyme has been observed (19–23). These aberrant fucosylations of the proteins and glycans are the potential biomarkers of cancers. α-1,6-Fucosyltransferase (core fucosyltransferase, FUT8) is highly expressed in many cancers but is decreased in gastric cancer (24–28). FUT8 is a driver of melanoma metastasis, which when FUT8 silenced, suppresses cell invasion and tumor dissemination were suppressed (28). Therefore, fucosyltransferase is a potential therapeutic target of cancers. In summary, fucosylation plays a pivotal role in proliferation, invasion, metastasis, and immune escape. Currently, many researchers are targeting the abnormality of fucosylation in lung cancer to identify potential biomarkers for diagnosis and treatment. In this article, the function of fucosylation in the progression of lung cancer is reviewed.

## Process of Fucosylation

**Figure 1:**
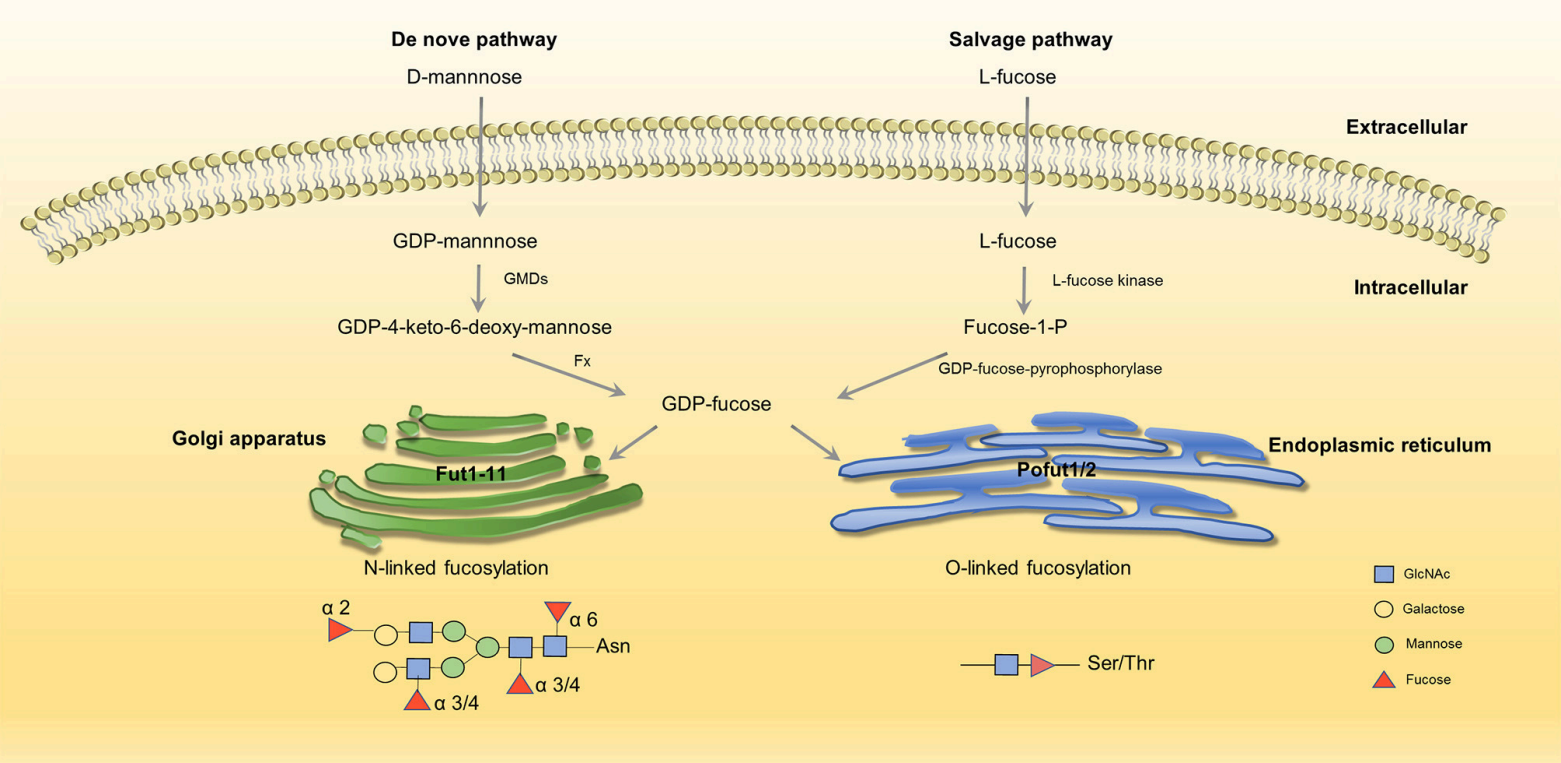
Two synthesis pathways of fucosylation. GDP-fucose is either synthesized from GDP-mannose (*de novo* pathway, left) or L-fucose (salvage pathway, right) before being transported into Golgi/ER through transporters (35, 36). No permissions were required to use the image.

Fucosylation is a typical terminal modification of proteins, and GDP-fucose is the only donor of fucosylation that is synthesized in the cytoplasm. GDP-fucose enters the endoplasmic reticulum or Golgi apparatus through the GDP-L-fucose transporter (GDP L-Fuc Tr) (29, 30). There are two synthesis pathways for the synthesis of GDP-fucose in mammals: *de novo* synthesis and salvage pathways. In the *de novo* synthesis pathway, GDP-mannose transforms into GDP-fucose, which is catalyzed by GDP-mannose-4,6-dehydratase (GMDS) and GDP-4-keto-6-deoxymannose-3,5-epimerase-4-reductase (Fx) (31, 32). In the salvage pathway, L-fucose transforms into GDP-fucose, which is catalyzed by L-fucose kinase and GDP-fucose pyrophosphorylase (33, 34) (Figure 1). Fucosylation is categorized according to the location of fucose. There are two kinds of *N*-fucosylation: core fucosylation (α-1,6-fucosylation) and terminal fucosylation (α-1,2-fucosylation, α-1,3/4-fucosylation). The participation of fucose is necessary for the formation of Lewis antigen. Lewis oligosaccharides have several structure types, such as Lewis a(Le^a^): Galβ1-3(Fucα-1,4) GlcNAc-R, sLewis a(sLe^a^): NeuAc2-3Galβ1-3(Fucα-1,4) GlcNAc-R, Lewis b(Le^b^): Fuc-1,2Galβ1-3(Fucα-1,4) GlcNAc-R, Lewis x(Le^x^): Galβ-1,4(Fucα-1,3) GlcNAc-R, sLweis x(sLe^x^): NeuAc-2,3Galβ-1,4(Fucα-1,3) GlcNAc-R, Lewis y(Le^y^): Fuc-1,2Galβ-1,4(Fucα-1,3) GlcNAc-R (35). Fucose residues are always involved in the synthesis of the terminal structure of glycans; thus, the transfer of the fucose residue to the glycan marks the end of glycan synthesis.

GDP-fucose serves as an essential substrate, and its synthesis is driven by GMDS and Fx. As a critical enzyme in fucosylation, the expression of GMDS is up-regulated in ADC at both mRNA and protein levels. In ADC, cell proliferation and survival are impaired when knockdown GMDS (37). Moreover, studies show that the abnormal expression of Fx is related to colitis, adenocarcinoma, colorectal cancer, and liver cancer (6, 38, 39)

Fucosyltransferases (FUTs) are enzymes that catalyze fucosylation. There are 13 types of FUTs. FUT1-11 exist in the Golgi apparatus and catalyze N-linked fucosylation, and O-fucosyltransferases (Pofuts) exist in the endoplasmic reticulum and catalyze O-linked fucosylation (35, 36, 40). FUT1 and FUT2 participate in the synthesis of α-1,2-fucosylation, FUT3,4,5,6,7,9 participate in α-1,3/4-fucosylation, FUT8 participates in α-1,6-fucosylation (41). Aberrant expression of FUTs occur in different kinds of cancer. FUT8 promotes breast cancer cell invasiveness by remodeling TGF-β receptor core fucosylation (21) and FUT4 is an effective biomarker for the diagnosis of breast cancer (42). We will explain in detail about the function and aberrant expression of FUTs below.

## Aberrant Fucosylation in Lung Cancer

### Aberrant Core Fucosylation in Lung Cancer

In mammals, core fucosylation is the primary type of N-fucosylation. The formation of α-1,6-fucosylation is in charge of Fut8, which is the only enzyme responsible for core fucosylation (43). Studies have confirmed that the activity and expression levels of Fut8 are significantly up-regulated in many malignant diseases, which reveal the involvement of Fut8 in the pathological process of these diseases (19, 44). Many studies have concentrated on the aberrant expression of core fucosylation in the sera and tissues of patients with lung cancer. Moreover, identifying the core fucosylated glycoproteins would contribute to confirming biomarkers of lung cancer. Lectin capture is the approach to acquire the core fucosylation glycoproteins, such as aleuria aurantia lectin (AAL), lens culinaris agglutinin (LCA), and pisum sativum agglutinin (PSA), which can recognize the core fucosylation structure specifically.

#### Aberrant Fucosyltransferase 8

Fut8 transfers a fucose moiety from GDP-β-L-fucose to the innermost GlcNAc residue in an N-glycan, and the resulting α1,6-fucose residue is designated as the core fucose (45). It has been implicated in several physiological and pathological processes. The function of the glycoproteins change due to altered fucosylation as a result of up-regulation of the α(1, 6)fucosyltransferase activity (21). Fut8 expression increased in the tissue of patients with stage I NSCLC, suggesting that Fut8 could be a prognostic factor. Moreover, silencing Fut8 could inhibit tumor growth and metastasis without affecting the proliferation of normal lung epithelial cells (19), indicating the association of high Fut8 expression with deterioration and poor survival. In NSCLC patients, Fut8 could be regarded as an unfavorable prognostic factor of potentially curatively resection, but it can be a potential therapeutic target for NSCLC (46) (Table 1).

**Table 1:** Overview of aberrant fucosylation in lung cancer.

**Lung cancer**	**Aberrant fucosylation**		**Effect**	**Clinical significance**	**References**
NSCLC	FUTs	FUT8	FUT8 increased in the NSCLC tissue	Potential biomarker	(21)
			Knockdown FUT8 could inhibit tumor growth and metastasis	Potential therapeutic target	(19)
		FUT2	FUT2 increased in the ADC tissue	Potential biomarker	(47)
			Knockdown FUT2 could inhibit cancer cell proliferation and metastasis, cell apoptosis increased	Potential therapeutic target	(48)
		FUT3	FUT3 activity was elevated significantly in the sera	Potential biomarker	(49, 50)
		FUT4	A strong expression of FUT4 exhibit a shorter survival period	Potential prognosis biomarker
		FUT7	A strong expression of FUT7 exhibit a shorter survival period	Potential prognosis biomarker
			Overexpression of FUT7 promote tumorigenesis improve EGFR/AKT/mTOR signaling pathway	Potential therapeutic target	(47)
	Fucosylated glycans	fucosylated glycans	Increased in ADC tissue	Potential biomarker	(51)
		Le^y^	The tyrosine phosphorylation of EGFR was inhibited	Potential biomarker	(52)
	Fucosylated proteins	AGP CP	Protein level express higher in ADC plasma	Potential biomarker	(53)
		C9	Fucosylation level of C9 express higher in SQCC	Potential biomarker	(54)
		HP	Fucosylation level of HP express higher in NSCLC	Potential biomarker	(55)
SCLC	FUTs	FUT1 FUT2	Increased in fucosyl GM1-positive SCLC cell lines	Potential biomarker	(56)
	fucosylated proteins	PON1	Protein level express lower in SCLC sera Fucosylation level of PON1 express higher in SCLC	Potential biomarker	(57)
		GM1	Have structure of α-1,2-fucosylated galactose, expressed in both SCLC cell lines	Application biomarker	(58)

*NSCLC, non-small cell lung cancer; SCLC, small cell lung cancer; FUTs, fucosyltransferases; AGP, alpha-1-acid glycoprotein; CP, ceruloplasmin; C9, protein complement component 9; HP, haptoglobin; PON1, paraoxonase 1; GM1, Monosialotetrahexosylganglioside*.

#### Aberrant Fucosylated Glycans and Proteins

E-cadherin was core fucosylated in highly metastatic lung cancer cells which is absent in lowly metastatic lung cancer cells (59). Ruhaak et al. used chip-based nano HPLC coupled with time-of flight mass spectrometry (nLC-chip-TOF-MS) to identify different glycan structures in ADC tissue. They described a detailed analysis of the differential N-glycosylation profile of the early stage lung adenocarcinoma tissue. The level of core fucosylated glycans increased in ADC compared with nonmalignant tissue (51), which may due to the increased expression of FUT8. Narayanasamy et al. found a type of upregulated protein complement component 9 (C9) in the sera of patients with SQCC using label-free liquid chromatography-electrospray ionization-tandem mass spectrometry (LC-ESI-MS/MS) analysis. The C9 protein level was 6.4-fold higher in SQCC patients compared to health control and C9 fucosylation levels were significantly higher (*p* < 0.05) in patients with SQCC (54), indicating that C9 and its core fucosylated form could serve as a useful marker for SQCC. Haptoglobin (HP) is a protein that is normally present in the blood that imparts antioxidant activity, promoting angiogenesis and other immune functions. HP is closely related to tumors, and the core fucosylation alteration of HP is also related to many diseases (60, 61). The level of HP fucosylation level was 2.55-fold higher in NSCLC patients compared to healthy controls, but no difference was detected in SCLC patients (55). Ahn et al. enriched the core fucosylated glycoproteins by AAL from patient sera. They identified four types of fucosylated proteins. Among them, the serum levels of paraoxonase 1 (PON1) were significantly reduced (< 0.77-fold) in patients with SCLC; nevertheless, the expression level of core fucose on PON1 was significantly increased (53). Studies have demonstrated that PON1 is related to many malignant diseases (62, 63). The core fucosylation level of PON1 and the specific glycan characteristics may serve as diagnostic and prognostic serological markers for SCLC.

Ahn et al. analyzed the core fucosylated proteins alpha-1-acid glycoprotein (AGP) and ceruloplasmin (CP) from ADC patients and healthy individuals using multiple reaction monitoring-mass spectrometry (MRM-MS). The core fucosylation level of AGP and CP were significantly higher in ADC patients compared to controls, although the levels of total plasma protein were comparable between the ADC and control groups (57). It is suggested that the aberrantly fucosylated target glycoproteins AGP and CP could be quantitatively identified as potential biomarkers of ADC. These results indicated that the level of core fucosylation, FUT8, and core-fucosylated products may provide new insights into the therapeutic targets of NSCLC (Table 1).

### Aberrant Terminal Fucosylation in Lung Cancer

FUT1-7 and FUT9-11 are responsible for the terminal fucosylation synthesis process. They are involved in the synthesis of Lewis antigen. α-1,2-Fucosylation is catalyzed by FUT1 and FUT2, α-1,3-fucosylation is catalyzed by FUT3–7 and FUT9, and α-1,4-fucosylation is catalyzed by FUT3 (64). The expression levels of sialyl Lewis x (sLe^x^), Lewis y (Le^y^), and sialyl Lewis a (sLe^a^) have significant variations in ADC, SQCC, and LC. In ADC, the expression levels of these Lewis antigens are the highest. Higher expression levels of these Lewis antigens decrease the survivability of the patients (65). Researches about terminal fucosylation in lung cancer mostly focus on α-1,2-fucosylation and α-1,3-fucosylation.

#### Aberrant Fucosyltransferase 1–7, 9–11

Studies have reported that miR-339-5p functions as a tumor suppressor in NSCLC (66). Because miR-339-5p can target FUT1 and regulate the downstream protein Le^y^, this could promote colony formation and attenuate apoptosis of lung carcinoma cell lines (67). The expression of FUT2 is higher in ADC than in the adjacent non-cancerous tissues. Knockdown of FUT2 in A549 and H1299 cells decreased the abilities of cell proliferation, migration, and invasion, but cell apoptosis increased compared with corresponding control cells. Moreover, FUT2 could enhance the cell migration and invasion of ADC cell lines and might promote ADC metastasis through the EMT initiated by TGF-β/Smad signaling. The results indicated that FUT2 is associated with ADC development and it would be a potential biomarker and therapeutic target of ADC (47, 48).

Many researchers have examined the role of α-1,3-fucosyltransferase (FUT3) in the synthesis of sLe^x^ and prognosis of its physiological function in lung cancer. The accumulation of tumor-associated antigen sLe^x^ was mostly studied in the sera of patients with lung cancer (68). sLe^x^ can serve as a ligand for the E- or P-selectin expressed on the cell surface, and its aberrant expression of sLe^x^ leads to cancer metastasis and angiogenesis. FUT3 activity was significantly elevated in the sera of patients with lung cancer compared to patients with benign diseases and healthy controls (49, 50). Ogawa et al. found that the sLe^x^ expression level was consistent with FUT4 and FUT7 expression. The survival period of the patients whose tumors showed a strong expression of FUT4 or FUT7 was significantly shorter than that of the patients whose tumors did not express either FUT4 or FUT7 (49). Overexpression of FUT7 promotes tumorigenesis in NSCLC by increasing the synthesis of sLe^x^. It activates the EGFR/AKT/mTOR signaling pathway which trigger cell proliferation (69). This may be the reason why patients who have a strong expression of FUT7 exhibit a shorter survival period. (Table 1).

#### Aberrant Fucosylated Glycans and Proteins

The tyrosine phosphorylation of epidermal growth factor receptor (EGFR) was inhibited upon Le^y^ was downregulated, which leads to the reduction of cell proliferation and tumor growth (52). Tsai et al. provided evidence of an increase in HP terminal fucosylation in NSCLC and SCLC through MS (70). Vasseur et al. found that levels of fucosylated tetra-antennary structures with varying degrees of sialylation are increased in the lung cancer patients who were former smokers. And the level of outer-arm fucosylation was elevated in the lung cancer samples provided by the former smokers (71).

Monosialotetrahexosylganglioside (GM1) is a type of ganglioside. Fucosyl GM1 has a unique structure of α-1,2-fucosylated galactose, which is expressed in both SCLC cell lines and tumor tissues (58). Fucosyl-GM1 has been considered a potential biomarker for SCLC. Tokuda et al. found increased expression of FUT1 and FUT2 in fucosyl GM1-positive SCLC cell lines, and FUT1 seemed to play a leading role in the fucosylation of GM1 in SCLC (56). The study of FUT1 and FUT2 provides novel insights into the role of fucosyl GM1 in novel therapies for SCLC (Table 1).

## Fucosylation and Proliferation, Invasion, and Metastasis

The proliferation, invasion, and metastasis of tumor cells are complex, and the cascade reactions mediated by alterations in the fucosylation patterns of the cell surface proteins (72–76). EMT is an important initiation reaction of the dissemination and metastasis of cancer cells (77). The process of EMT is associated with the alteration in some molecular markers, such as E-cadherin, plakoglobin, and cytokeratins. E-cadherin is a type of epithelial markers that shows lower expression in EMT, leading to impairment of cell-cell adhesion, which allows detachment of cells (78). Tumor cells acquire the ability to invade and migrate when E-cadherin is suppressed, which could lead to tumor progression and metastasis (79, 80). The glycosylation of E-cadherin plays a critical role in the regulation of tumor metastasis (81). The fucosylation level of E-cadherin and its effect on lung cancer were studied.

**Figure 2:**
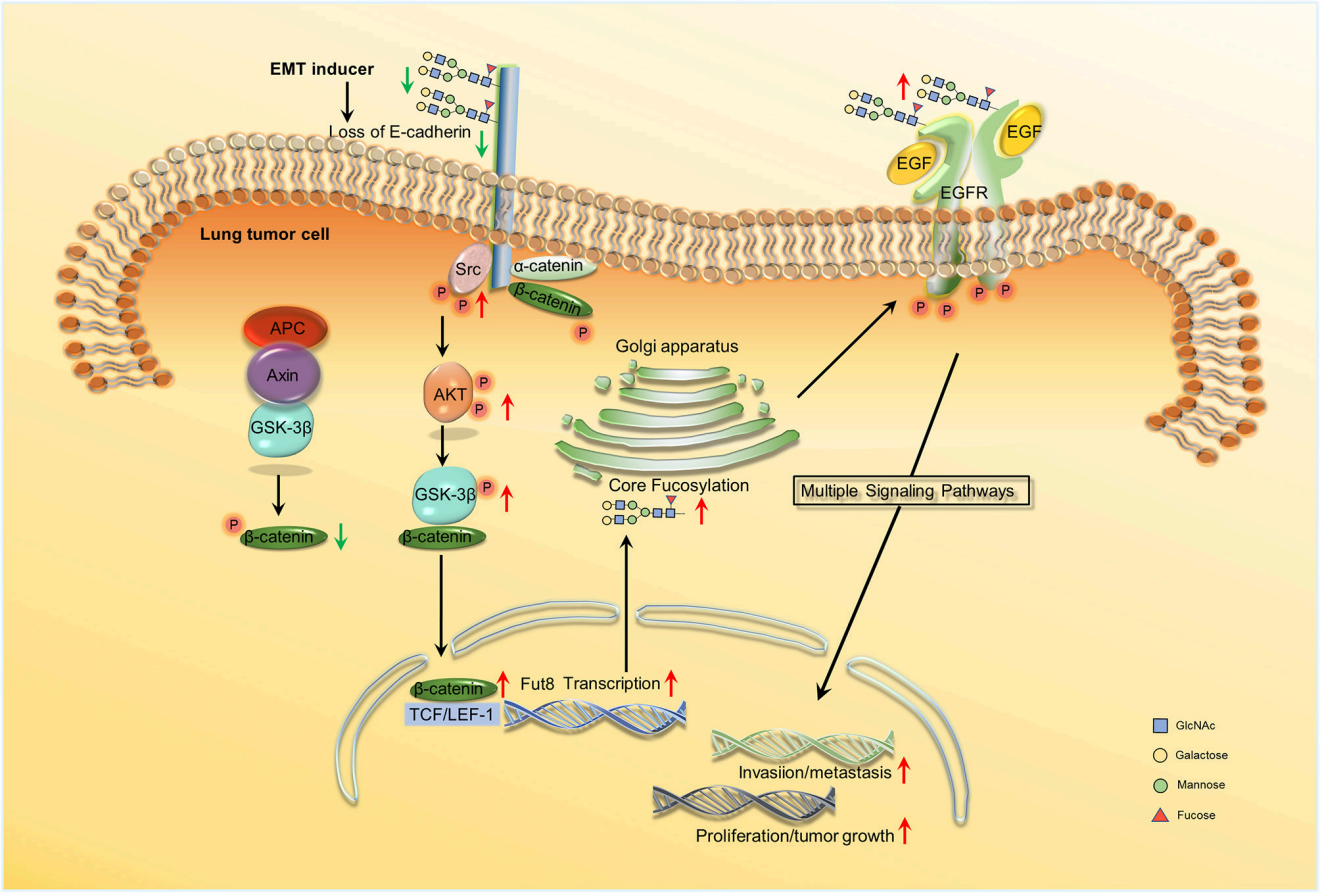
Schema of core fucosylation regulation of lung cancer progression. A decreased level of core fucosylation of E-cadherin activates Src, which leads to the accumulation of nuclear β-catenin (83, 85). β-catenin can further combine with LEF1 and promote the transcription of Fut8. Besides, the upregulation of core fucosylation will result in the dimerization and phosphorylation of EGFR and ultimately activate the downstream signaling and the cascade reactions (86, 19). No permissions were required to use the image.

Wen et al. showed that in ADC progression, the core fucosylation of E-cadherin is increased (82). Geng et al. observed that core fucosylated E-cadherin was prominently expressed in highly metastatic lung cancer cells, while there was almost no expression in cells with low levels of metastasis (59). They observed that when E-cadherin was combined with reduction of core fucosylation, cell–cell adhesion was strengthened. However, cell–cell adhesion weakened when the elevated core fucosylated on E-cadherin. Hu et al. then found that core fucosylated E-cadherin is related to the accumulation of nuclear β-catenin in lung cancer cells. If E-cadherin was core-fucosylated, the nuclear β-catenin was reduced; the opposite occurs upon reduced core fucosylation on E-cadherin (83). Shao et al. explained the mechanism of the results above. They revealed the regulation mechanism of core fucosylated E-cadherin in lung cancer cells. They observed a decreased expression of E-cadherin and its core fucosylation using immunohistochemistry (IHC) in lung tumor tissues. Since the core fucosylated E-cadherin regulated the activity of Src and β-catenin, enhanced levels of phosphorylated β-catenin and Src were observed upon lower core fucosylation on E-cadherin. The Src kinase then induces AKT phosphorylation and the activated AKT in turn phosphorylated GSK-3β. GSK-3β is considered to form a β-catenin-degradation complex, which bind to β-catenin, axin, and APC (adenomatous polyposis coli) (84). The activity of GSK-3β was inhibited after phosphorylation, and the inactivated GSK-3β was then unable to phosphorylate β-catenin. Ubiquitylation and degradation of the β-catenin-degradation complex is inhibited. A high expression of phosphorylated GSK-3β then led to increasing nuclear β-catenin accumulation. This is the reason why the accumulated nuclear β-catenin was observed to increase if the core fucosylation of E-cadherin reduced (85). Core fucosylation could not only affects the dimerization of E-cadherin, but also promotes intracellular combination with catenins; core fucosylation on E-cadherin could then regulate the molecular function and its downstream signaling (83). During the process of EMT in lung cancer cells, the downregulation of E-cadherin leads to an increased accumulation of nuclear β-catenin. The nuclear β-catenin could then specifically bind to lymphoid enhancer factor-1 (LEF-1) transcription factors to activate FUT8 transcription. The increase in core fucose synthesis, for example in EGFR, occurs due to FUT8 upregulation. Increasing sialylation and α-1,3-linked fucosylation suppresses EGFR dimerization and phosphorylation, whereas increasing α-1,6-linked fucosylation promotes dimerization and phosphorylation of EGFR (86). Dimerized and phosphorylated EGFR then promotes its downstream signaling pathway, which promotes tumor cell proliferation, invasion, and metastasis by activating the expression of genes involved in cell adhesion, motility, growth, and angiogenesis (19) (Figure 2). In addition, terminal fucosylation plays a critical role in lung cancer cell proliferation, invasion, and metastasis. Liang et al. studied the molecular and biological mechanisms of FUT7 elevation in lung cancer (69). Farhan et al. showed that EGFR signaling was inhibited by anti-Lewis^y^ antibodies in A431 cells, which could lead to the inhibition of cell proliferation (87).

## Discussion

The aberrant fucosylation of tumor is always involved in the activation of EGFR or TGF-βR, and affect the function of integrins, selectins, and apoptotic signaling pathways; ultimately influencing tumor proliferation, invasion, apoptosis, and metastasis. The fucosylation is different in different tumors, different pathological types, and different developmental stages of a tumor. Abnormal expression of fucosylated glycans and proteins were observed in the sera or tissues of patients with lung cancer. In summary, we found that FUTs play an important role in the regulation of fucosylation. Thus, the aberrant expression of FUTs can be considered as clinical biomarkers and therapy target improve the survival rate. FUT8 has received considerable attention because of its absolute specificity. That is why studies about aberrant terminal fucosylated proteins in lung cancer are less frequent, and the terminal structures are characterized by complexity and diversity. Although less is known about the fucosylation in SCLC, it is characterized by more higher malignancy, more often remote metastasis and poor prognosis. The current information might provide us research direction for SCLC. It is significant to pay more attention to SCLC and glycosylation (Table 1).

In the past decades, numerous alterations in fucosylation have been considered as potential biomarkers of lung cancer. The deregulation of fucosylation in cancer and its contribution to tumor–microenvironment interactions remain poorly understood. Moreover, studies have mainly focus on *N*-fucosylation with little concern about *O*-fucosylation. Currently, most studies have concentrated on the relationship between POFUT and the Notch pathway (88, 89). The deletion of the *O*-fucosylation-related *Pofut1* gene leads to the failure of Notch binding to the Notch ligands, and thus, blocks signal transmission (90). As the Notch signal is responsible for cell proliferation and apoptosis, POFUT1 may participate in cancer progression.

In contrast, fucosidase, a hydrolase of fucose plays an important role in some malignant diseases. α-L-fucosidase 1 (FUCA1) is a lysosomal enzyme, which reverses the role of FUTs. α-L-fucosidase 1 is responsible for catalyzing the hydrolysis of the terminal fucose residue and plays a crucial role in the breakdown of fucose-containing glycoproteins and glycolipids (91). Cheng et al. reported that the expression of FUCA1 mRNA was increased in breast cancer samples when compared with normal tissue samples. In addition, FUCA1 levels are shown to be higher in advanced-stage tumors. However, the levels of fucosylated Lewis-x antigen are shown to be lower in the advanced-stage patients. Cheng et al. proposed that FUCA1 may be used as a potential prognostic molecular target for breast cancer in clinical investigations (92). In contrast, other researchers have confirmed that FUCA1 pretreatment significantly reduces the invasive capacity of MDA-MB-231 breast cancer cells (93). Vecchio et al. reported that the decreased expression of FUCA1 is related to the augmentation of thyroid cancer, which indicated that FUCA1 levels could be considered reliable evidence for medical diagnoses for aggressive thyroid cancer (94). There is little knowledge on the relationship between FUCA1 and lung cancer. Therefore, we hypothesized that the upregulation of FUCA1 promotes fucose-containing molecules to downregulate their expression on the surface of cancer cells, which can significantly inhibit tumor cell invasion.

Besides fucosylation, other glycosylation types are also closely connected with lung cancer. *O*-GlcNAcylation and *O*-GlcNAc transferase (OGT) expression was significantly elevated in lung cancer tissues. *O*-GlcNAcylation could also enhance lung cancer invasion (95). That the expression of sialyltransferase was up-regulated in lung cancer cell and inhibited the phosphorylation and the dimerization of EGFR (86). Mucins are high molecular weight, heavily glycosylated proteins expressed in the epithelial cells of various organs, are characterized by extensive *O*-glycosylation. Several reports suggest that mucins are overexpressed in NSCLC and can be used as suitable biomarkers for identifying and monitoring the progression of lung cancer (96, 97).

We found that these aberrant glycosylations lead to disorder of the proliferation, apoptosis, invasion, and metastasis in lung tumor cells, which in common with fucosylation. The difference between these patterns is that the glycosylation modification is different in different proteins, which is involved in the process of lung cancer. These proteins function in tumor cells metabolism. Therefore, there is plenty of scope for further exploration in this field.

## Conclusion

This review aimed to improve the overall understanding of the relationship between lung cancer and fucosylation. Lung cancer is a common problem and a major cause of morbidity and mortality. Using molecular biology and glycomics approaches, we will have a better understanding in the pathophysiological features of lung cancer. They provide us new ideas about the development of novel biomarkers for early diagnosis and targeted therapy. We hope that increased awareness of up-to-date evidence-based knowledge and targeting the abnormality of fucosylation in lung cancer will become a new strategy.

## Author Contributions

All authors participated in literature research and data classification. LJ, TM, and YG wrote the manuscript. JZ, YY, and JC reviewed and edited the manuscript before submission.

### Conflict of Interest Statement

The authors declare that the research was conducted in the absence of any commercial or financial relationships that could be construed as a potential conflict of interest.

## Abbreviations

NSCLC: non-small cell carcinoma
SCLC: small cell carcinoma
ADC, adenocarcinomas, SQCC: squamous cell carcinomas
LC: large cell carcinomas
PTM: post translationally modified
FUCA-1: α-L-fucosidase-1
PTC: papillary thyroid carcinomas
ATC: anaplastic thyroid carcinomas
EMT: epithelial–mesenchymal transition
sLe^x^: sialyl Lewis x
Le^y^: Lewis y
sLe^a^: sialyl Lewis a
EGFR: epidermal growth factor receptor
FUCA1: α-L-fucosidase 1.
